# Causal effects between gut microbiota and IgA nephropathy: a bidirectional Mendelian randomization study

**DOI:** 10.3389/fcimb.2023.1171517

**Published:** 2023-05-02

**Authors:** Feihong Ren, Qiubai Jin, Tongtong Liu, Xuelei Ren, Yongli Zhan

**Affiliations:** ^1^ Guang’anmen Hospital, China Academy of Chinese Medical Sciences, Beijing, China; ^2^ Graduate School, Beijing University of Chinese Medicine, Beijing, China

**Keywords:** two-sample mendelian randomization, Bayesian model averaging, gut microbiota, IgA nephropathy, pathogenesis, causality

## Abstract

**Background:**

Therapeutic approaches that target the gut microbiota (GM) may be helpful in the potential prevention and treatment of IgA nephropathy (IgAN). Meanwhile, relevant studies demonstrated a correlation between GM and IgAN, however, these confounding evidence cannot prove a causal relationship between GM and IgAN.

**Methods:**

Based on the data from the GM genome-wide association study (GWAS) of MiBioGen and the IgAN GWAS data from the FinnGen research. A bi-directional Mendelian randomization (MR) study was performed to explore the causal relationship between GM and IgAN. We used inverse variance weighted (IVW) method as the primary method to determine the causal relationship between exposure and outcome in our MR study. Besides, we used additional analysis (MR-Egger, weighted median) and sensitivity analysis (Cochrane’s Q test, MR-Egger and MR-PRESSO) to select significant results, followed by Bayesian model averaging (MR-BMA) to test the results of MR study. Finally, a reverse MR analysis was conducted to estimate the probability of reverse causality.

**Results:**

At the locus-wide significance level, the results of IVW method and additional analysis showed that Genus Enterorhabdus was a protective factor for IgAN [OR: 0.456, 95% CI: 0.238-0.875, p=0.023], while Genus butyricicoccus was a risk factor for IgAN [OR: 3.471, 95% CI: 1.671-7.209, p=0.0008]. In the sensitivity analysis, no significant pleiotropy or heterogeneity of the results was found.

**Conclusion:**

Our study revealed the causal relationship between GM and IgAN, and expanded the variety of bacterial taxa causally related to IgAN. These bacterial taxa could become novel biomarkers to facilitate the development of targeted therapies for IgAN, developing our understanding of the “gut-kidney axis”.

## 1 Introduction

IgA nephropathy (IgAN), regarded as an immune-complex-mediated glomerulonephritis (Wang et al., 2020), is characterized by pathological features including granular deposition of IgA (Gd-IgA1) in the glomerular mesangial areas (Suzuki et al., 2011), the proliferation of glomerular mesangial cells and increases in the mesangial matrices (Levy and Berger, 1988). IgAN is the most common primary glomerular disease worldwide (Schena and Nistor, 2018), a significant feature of this nephropathy is the relatively poor outcome, which is a common cause of End-stage renal disease (ESRD) in young people (D'Amico, 2000; Lee et al., 2014). Although kidney transplantation is available for IgAN patients, the postoperative recurrence rate can be as high as 50% (Ponticelli and Glassock, 2010; Uffing et al., 2021), so most patients prefer to rely on regular hemodialysis as a maintenance treatment (Chawla et al., 2014). Furthermore, the disease occurs mainly in young and middle-aged people, who represent the main force of society (McGrogan et al., 2011). All these factors not only bring mental and economic pressure to patients and families, but also have a certain impact on the economic development of society (Sallustio et al., 2019). Therefore, researchers need to conduct a comprehensive search for risk factors affecting the disease, which assist in the prevention and treatment of IgAN.

In recent years, increasing evidence indicated that abnormalities of the intestinal mucosal immune system are closely associated with the pathogenesis of IgA nephropathy. Rosanna (Coppo, 2015) suggested that when the immune function of the intestinal mucosa becomes abnormal, a deficiency in immune tolerance to GM occurs in the organism, which leads to a decrease in intestinal barrier function, and an increase in blood entry of intestinal endotoxins, leaving the body in an inflammatory state. As a result, a large amount of Gd-IgA1 is produced, and this leads to the development of IgAN. Additionally, GM can also affect the development of IgAN by repairing or damaging the intestinal mucosal barrier (Camilleri et al., 2012). All the above studies suggest that gut microbiota impacts the development of IgAN in different ways.

Bacteria in the human intestine are widely distributed, with approximately 200-500 species and 100 trillion in number (Kåhrström et al., 2016), all of which work together to form the human gut microbiota (GM). The GM contains both beneficial and potentially pathogenic bacteria, which include a dynamically balanced symbiosis with the organism. Therefore, GM has an essential role in maintaining the immune homeostasis of the organism and resisting enteric-derived toxins, such as controlling the differentiation of immune cells or maintaining the intestinal mucosal barrier function by promoting the expression of tight junction proteins in intestinal epithelial cells and the secretion of antimicrobial peptide substances. A cross-sectional study confirmed (De Angelis et al., 2014) that GM showed differences between IgAN patients and normal healthy individuals, whereby patients had a significant reduction of beneficial bacteria in their stools and a significant increase of harmful bacteria compared to normal healthy individuals. Additionally, studies (De Angelis et al., 2014) on mice that were transfected with B cell activation factor of the TNF family (BAFF) overexpression gene indicated that dysbiosis of GM may promote the development of IgAN (De Angelis et al., 2014). However, traditional epidemiological studies can be susceptible to confounding factors, study sample size and reverse causality. Thus, a causal relationship between GM and IgAN risk is not reliably inferred from the observed results. It can be stated that the causal relationship between GM and IgAN is still unclear. So, it is necessary to comprehensively assess the causal relationship between GM and IgAN from the genetic level.

Mendelian randomization (MR) analysis is a new genetic statistical method that can be used as an effective alternative to traditional epidemiological research methods. It uses genetic variants strongly associated with exposure factors as instrumental variables (IVs) to evaluate a statistical causal relationship between exposure and outcome (Katan, 1986; Smith and Ebrahim, 2003; Davies et al., 2018). The advantage of MR over traditional epidemiological research methods is that the random assignment method is determined by DNA genotype, which can limit the influence of external factors on the robustness of causality to the greatest extent possible (Ebrahim and Davey Smith, 2008; Emdin et al., 2017). Furthermore, genotype formation precedes disease and is typically unaffected by disease progression, which is why Mendelian randomization studies can also avoid reverse causality (Soremekun et al., 2022).

To comprehensively explore the potential GM associated with IgAN, this study used the latest GM genome-wide association study (GWAS) data to identify novel factors causally associated with IgAN, which could become potential biomarkers to predict the development of IgAN. In conclusion, the findings of this work can provide more information than a single biological signal and can also offer assistance in the development of IgAN screening and prevention.

## 2 Materials and methods

### 2.1 Study design

**Figure 1:**
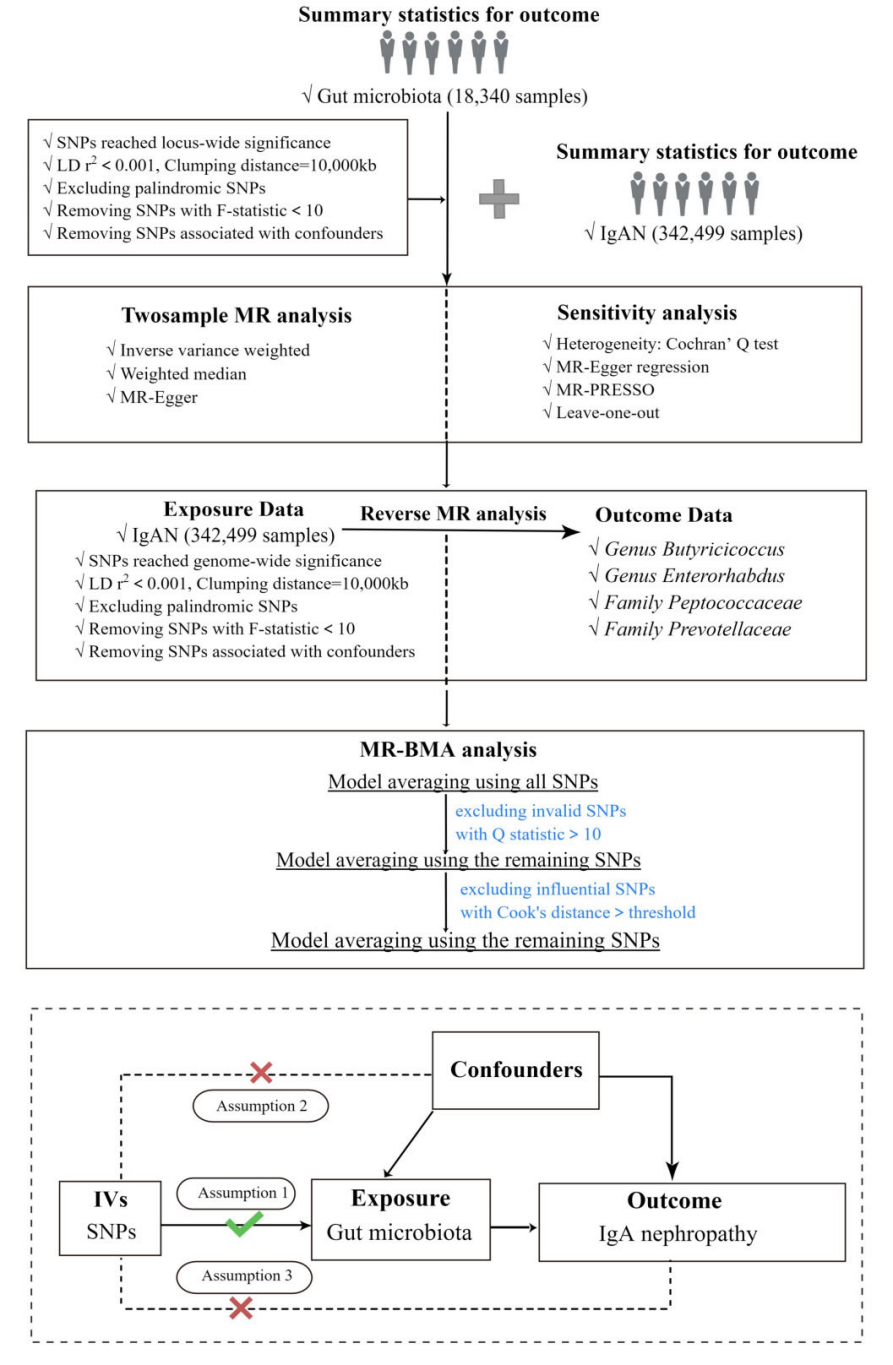
The study design and major assumptions of the Two-sample MR study. LD r^2^, r^2^ in linkage disequilibrium; SNPs, single nucleotide polymorphisms; IgAN, IgA nephropathy; MR-PRESSO, Mendelian Randomization Pleiotropy RESidual Sum and Outlier; IV, instruments variable; MR-BMA, MR Bayesian Model Averaging.

In the current study, two-sample Mendelian Randomization was used to assess the relationship between GM and IgAN, and the overall study flow is shown in **Figure 1**. Three basic assumptions must be satisfied in order to ensure the reliability of MR results: (I) the instrumental variables (IVs) used in the analysis are significantly correlated with exposure. (II) the IVs are independent of confounders that influence exposure and outcome. (III) the IVs affect the outcome only through exposure but not via other pathways (Davey Smith and Hemani, 2014). Among these, both the second and third hypotheses are designed to ensure that the study is independent, which can be tested by a series of statistical methods.

### 2.2 Mendelian randomization analysis

In this study, the Inverse Variance Weighted (IVW) was the major method to infer the causal relationship between GM and IgAN when no horizontal pleiotropy was present (Burgess et al., 2013). Additionally, the Weighted Median method (WM) and MR-Egger method were employed as additional methods to IVW in order to obtain more robust results. The additional methods have to meet the assumptions of their respective models: the WM method assumes that at least half of the single nucleotide polymorphisms (SNPs) are free of pleiotropy (Hartwig et al., 2017); if the number of SNPs with pleiotropy exceeds 50%, the results of MR-Egger inference are still robust (Bowden et al., 2015). As for the significance of the study results, this study defined that a causal relationship between exposure and outcome was considered to exist when the IVW_P-value_ was less than 0.05, which we defined as achieving nominal significance; if the results were supported by one and more additional methods, they were considered to be significant (Ni et al., 2021).

### 2.3 Mendelian randomization Bayesian model averaging

Since the causal GMs have shown high correlations in terms of sharing a large number of genetic variants, it was necessary to correct the influence of “measured polymorphism”. Thus, we applied Bayesian model averaging (BMA) to further validate the results of IVW_P-value_<0.05 (Wang et al., 2022). In addition, the average causal effect of the models generating IgAN according to the MIP ranking can be used to generate comparisons of risk factors or to explain the direction of impact. Ultimately, the best models were selected preferentially based on the PP values of the independent models (threshold of 0.02), and we excluded Family Prevotellaceae (PP=0.014) and Genus Butyricicoccus (PP=0.009). We identified and eliminated outliers, i.e. SNPs with Q-statistic value > 10 and SNPs whose Cook’s distance exceeded the threshold. The analysis was repeated after the outliers were removed until no outliers were found.

### 2.4 Reverse-direction mendelian randomization analysis

In addition, we performed reverse Mendelian Randomization of bacterial taxa supported by the IVW method to test whether there was a causal effect of IgAN on GM. Notably, we applied reverse Mendelian randomization analyses between IgAN and specific causal bacterial taxa, the reverse causal relationships between them were not found. The steps of reverse Mendelian Randomization were the same as those of Mendelian Randomization, as shown in **Figure 1**.

### 2.5 Sensitivity analysis

Influenced by the diversity of study subjects and research methods, heterogeneity may exist in Mendelian Randomization analysis. To avoid the effect of heterogeneity on the causal effect, Cochran’s Q tests were conducted to detect heterogeneity within the instrumental variables (IVs). The impact of heterogeneity on the causal effect could be ignored if p>0.05; on the other hand, if there was significant heterogeneity (p< 0.05), the IVW-random effects model was employed to reduce the effect of heterogeneity on the causal effect. Besides, considering the impact of unknown confounders on genetic diversity and causal effects, we used MR-Egger regression to evaluate whether the included SNPs had potential horizontal pleiotropy, and results with horizontal pleiotropy (p< 0.05) would be excluded (Bowden et al., 2015). Besides, Mendelian Randomization pleiotropy residual sum and outlier (MR-PRESSO) was further applied to detect any outlier reflecting polymorphic deviations due to the lower accuracy and statistical efficacy of MR-Egger regression. After excluding outliers, MR analysis was re-performed, and causal effects were re-given (Verbanck et al., 2018). All re-analysis results from this study did not change significantly. The results of sensitivity analyses for all IVs are shown in **Supplementary Table 1**. In addition, we performed the leave-one-out sensitivity analysis to assess whether the causal association of the MR analysis was caused by a single IV (Xiang et al., 2021). The results were considered reliable if the overall error line did not change considerably after excluding SNPs one by one. As for outliers defined in the leave-one-out analyses, we defined outliers as those observations whose Cook’s distance exceeded a certain threshold (0.5 in our case).

### 2.6 Data sources

Summary-level GWAS data for human GM were obtained from the MiBioGen consortium, and data were derived from 18,340 European ethnic participants from 24 cohorts in 11 countries. The GWAS data could be obtained at https://mibiogen.gcc.rug.nl/. This is the largest genome-wide meta-analysis of GM conducted to date (Kurilshikov et al., 2021). From 211 bacterial taxa in total, this study ultimately identified 122,110 variant sites at five levels: phylum, class, order, family, and genus. To ensure the accuracy of the data, our study excluded 15 bacterial taxa of unknown family or genus, leaving 196. The GWAS data for IgA nephropathy (IgAN) were obtained from the FinnGen research, which included 538 cases and 341,961 controls from the Finnish population. In FinnGen research, the International Classification of Diseases (ICD) diagnosis codes N082&D8980 used to define IgAN. The GWAS data could be obtained at https://r8.finngen.fi/.

### 2.7 Selection of instruments variables

To obtain qualified instrumental variables (IVs), we performed quality checks on single nucleotide polymorphism (SNP): (I) As the number of SNPs included under the genome-wide significance threshold (p< 5×10^-8^) was too limited, which may lead to a loss of potentially meaningful results. We used the locus-wide significance threshold (p< 1×10^-5^) to select SNPs that were associated with exposure, with the locus-wide significance P-value threshold having been widely used in previous Mendelian Randomization studies (Wang et al., 2021). (II) To ensure no linkage disequilibrium between IVs associated with GM, we performed clumping processes (r^2^< 0.001, Clumping distance = 10000kb) on the screened SNPs to retain independent SNPs. (III) When some SNPs were not in the outcome GWAS, we looked for proxy SNPs that shared high level of linkage disequilibrium (R^2^>0.8) with the target SNPs. (IV) The palindromic SNPs were excluded to make sure that the effects of SNPs on exposure matched the effects on outcome that correspond to the same alleles. (V) To avoid SNPs being associated with potential risk factors on outcomes, the Phenoscanner was used to examine and screen out SNPs associated with potential confounders or risk factors (hypertension, diabetes, obesity, etc.). (VI) To prevent bias caused by weak IVs, we calculate the F-statistic for each bacterial taxon using the following formula:


$$\text{F=}\frac{{\text{R}}^{2}\times (n-k-1)}{\text{k}\times {\text{(1-R}}^{2})}$$

R ^2^ is the exposure variance explained by the selected SNPs, n represents the sample size, and k represents the number of included instrumental variables (Xu et al., 2021; Li et al., 2022). IVs with F-statistics less than 10 were considered weak and excluded (Burgess and Thompson, 2011).

### 2.8 Statistical analysis

All statistical analyses in this study were performed by the “TwoSampleMR” package in the R program (Version 4.2.2). The MR-PRESSO test was performed by the “MR-PRESSO” package. Figdraw (www.figdraw.com) was used for the graphical plotting in this study.

### 2.9 Ethics

The summary-level data used in this study were publicly available de-identified data, which were approved by the Ethical Standards Committee. No separate ethical approval was required for this study.

## 3 Results

### 3.1 Selection of IVs related to GM

**Figure 2:**
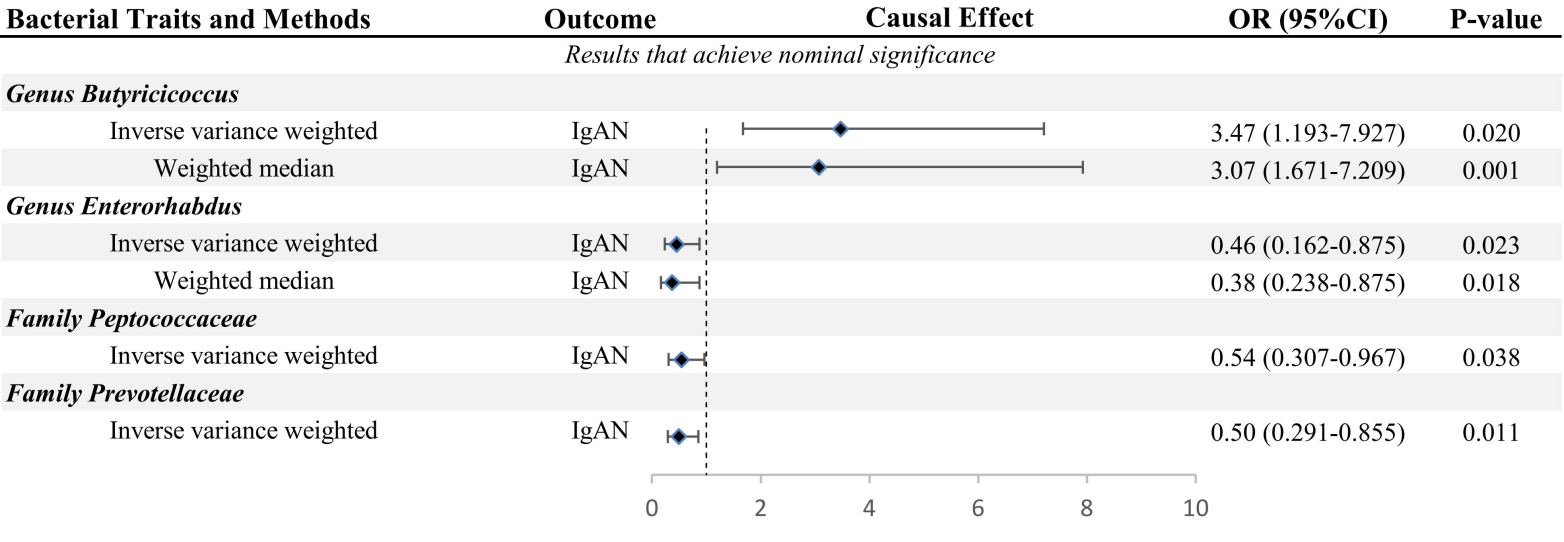
MR results of GM taxa with nominal significance causal relationships to IgAN.

In this study, we used the locus-wide significance threshold to screen SNPs and removed SNPs with linkage disequilibrium effect, and 2,604 SNPs were retained. Then, after eliminating SNPs that might be associated with confounding factors, we included 2,538 snps to predict IgAN genetically (**Supplementary Table 2**). Ultimately, bacteria taxa with nominal significance were identified (**Table 1** and **Figure 2**). However, only Genus Butyricicoccus and Genus Enterorhabdus showed significance after additional MR analysis and sensitivity analysis.

**Table 1:** Significant MR analysis results of causal links between GM and IgAN.

Gut microbiota	Nsnps	Traits	Method	OR	OR (95% CI)	Beta	*p*-value	MR-Egger Regression		Heterogeneity (IVW)	
								Egger Intercept	*p*-value	Cochran’s Q	*p*-value
Genus Butyricicoccus	8	IgAN	MR-egger	3.209	0.766-13.455	1.166	0.162	0.008	0.905	3.836	0.798
Genus Butyricicoccus	8	IgAN	WM	3.075	1.193-7.927	1.123	0.020				
Genus Butyricicoccus	8	IgAN	IVW	3.471	1.671-7.209	1.244	0.001				
Genus Enterorhabdus	6	IgAN	MR-egger	0.307	0.055-1.713	-1.181	0.249	0.056	0.652	2.358	0.798
Genus Enterorhabdus	6	IgAN	WM	0.377	0.162-0.875	-0.976	0.023				
Genus Enterorhabdus	6	IgAN	IVW	0.456	0.238-0.875	-0.785	0.018				
Family Peptococcaceae	9	IgAN	MR-egger	1.480	0.300-7.296	0.392	0.645	-0.097	0.229	6.180	0.627
Family Peptococcaceae	9	IgAN	WM	0.569	0.261-1.238	-0.565	0.155				
Family Peptococcaceae	9	IgAN	IVW	0.545	0.307-0.967	-0.608	0.038				
Family Prevotellaceae	16	IgAN	MR-egger	0.242	0.034-1.709	-1.420	0.177	0.052	0.462	8.230	0.914
Family Prevotellaceae	16	IgAN	WM	0.559	0.276-1.130	-0.582	0.105				
Family Prevotellaceae	16	IgAN	IVW	0.499	0.291-0.855	-0.695	0.011				

Nsnps, number of single nucleotide polymorphism; IgAN, IgA nephropathy; OR, odds ratio; IVW, inverse variance weighted method; WM, weighted median method.

### 3.2 Causal effects and sensitivity analysis of GM on IgAN

**Figure 3:**
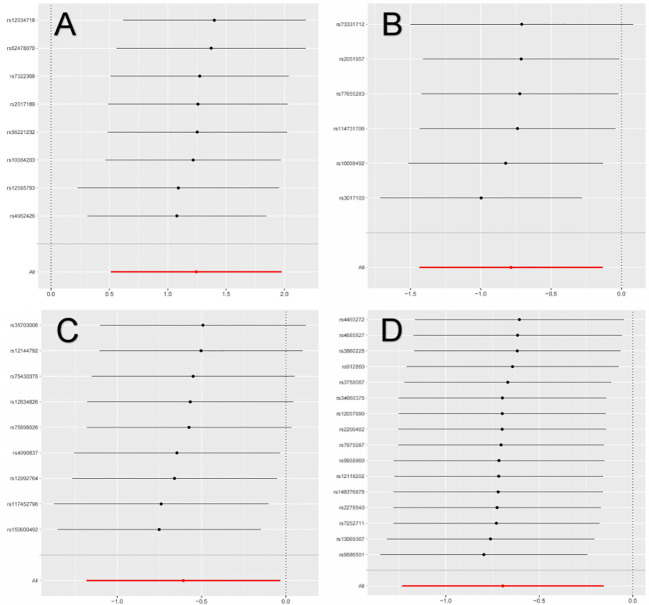
Leave-one-out analysis of the causal association between GM and IgAN. **(A)** Leave-one-out analysis of the causal association between Butyricicoccus and IgAN; **(B)** Leave-one-out analysis of the causal association between Enterorhabdus and IgAN; **(C)** Leave-one-out analysis of the causal association between Peptococcaceae and IgAN; **(D)** Leave-one-out analysis of the causal association between Prevotellaceae and IgAN.

The primary MR analysis supported the association of four bacterial taxa with the risk of IgAN, with all the IVs meeting the requirements of strong instrumental variables (F > 10), as shown in **Table 1**. However, further additional analyses and sensitivity analyses only considered the results of two bacterial taxa to be significant. Specifically, a higher abundance of Family Peptococcaceae, Family Prevotellaceae, and Genus Enterorhabdus were associated with a lower risk of IgAN [odds ratio (OR): 0.545, 95% confidence interval (CI): 0.307-0.967, p=0.038 for Family Peptococcaceae; and OR: 0.499, 95% CI: 0.291-0.855, p=0.011 for Family Prevotellaceae, and OR: 0.456, 95% CI: 0.238-0.875, p=0.023 for Genus Enterorhabdus]. Conversely, a higher abundance of Genus Butyricicoccus was related to a higher risk of IgAN [OR: 3.471, 95% CI: 1.671-7.209, p=0.0008], as shown in **Supplementary Table 3** and **Figure 2**. In sensitivity analyses, no evidence of horizontal pleiotropy was indicated by the results of MR-Egger regression intercepts (p>0.05), as shown in **Supplementary Table 1**. The results of the MR-Egger regression were further validated by using MR-PRESSO, which showed no evidence of outliers (**Supplementary Table 1**). The leave-one-out method did not suggest significant outliers in **Figures 3A, D**. Abnormal values were observed in **Figures 3B, C**, however, no other statistical methods (MR-PRESSO global test and MR-Egger intercept test) showed anomalies, which confirmed that these outliers did not significantly influence the results. In summary, most of the sensitivity analyses indicated that the results of the MR analysis of the study were reliable.

### 3.3 MR-BMA method to identify leading taxa on IgAN

We applied the MR-BMA method to rank Genus Enterorhabdus, Family Peptococcaceae, Family Prevotellaceae, and Genus Butyricicoccus, and included the remaining 40 SNPs in the analysis after excluding rs11098863 whose Cook’s distance exceeded the threshold. In the MR-BMA method, we calculated the posterior probability (PP) for each model. We ranked bacterial taxa from most to least important using the marginal inclusion probability (MIP), which is the sum of the PPs of all possible models. The highest MIP rank indicated the strongest “true causal” association candidate (Zuber et al., 2020; Lord et al., 2021). Notably, MR-BMA results showed that the abundance of Genus Enterorhabdus was negatively correlated with the risk of IgAN. After correction for outliers, we repeated the analysis and found that Genus Enterorhabdus played a dominant role in the risk of IgAN (**Table 2**).

**Table 2:** Ranking of gut microbiota for IgAN using MR-BMA.

Model averaging employing 41 SNPs (Q-statistics indicated no deviant instruments (all Q< 10)							
Risk factor or model	MIP	Rank by MIP	MACE	PP	Rank by PP	Causal estimates	p
Enterorhabdus	0.705	1	-0.398	0.444	1	-0.569	0.02970297
Peptococcaceae	0.329	2	-0.13	0.167	2	-0.418	0.12871287
Prevotellaceae	0.176	3	-0.058	0.063	3	-0.329	0.84158416
Butyricicoccus	0.103	4	-0.009	0.038	4	-0.087	0.99009901
Model averaging employing 40 SNPs (excluding influential instrument rs11098863 with Cook’s distance exceeding the threshold)							
Risk factor or model	MIP	Rank by MIP	MACE	PP	Rank by PP	Causal estimates	p
Enterorhabdus	0.929	1	-0.689	0.563	1	-0.747	0.00990099
Peptococcaceae	0.252	2	-0.095	0.041	2	-0.423	0.40594059

SNPs, single nucleotide polymorphisms; MIP, marginal inclusion probability; MACE, model-average causal effect; MR-BMA, MR based on Bayesian model averaging; PP, posterior probability.

### 3.4 Causal effects and sensitivity analysis of IgAN on GM

Finally, we performed reverse Mendelian Randomization on the results reaching nominal significance to test whether the genetically predicted IgAN would be causal for GM. However, significant results were not found in the reverse MR analysis, as shown in **Supplementary Table 4**.

## 4 Discussion

**Figure 4:**
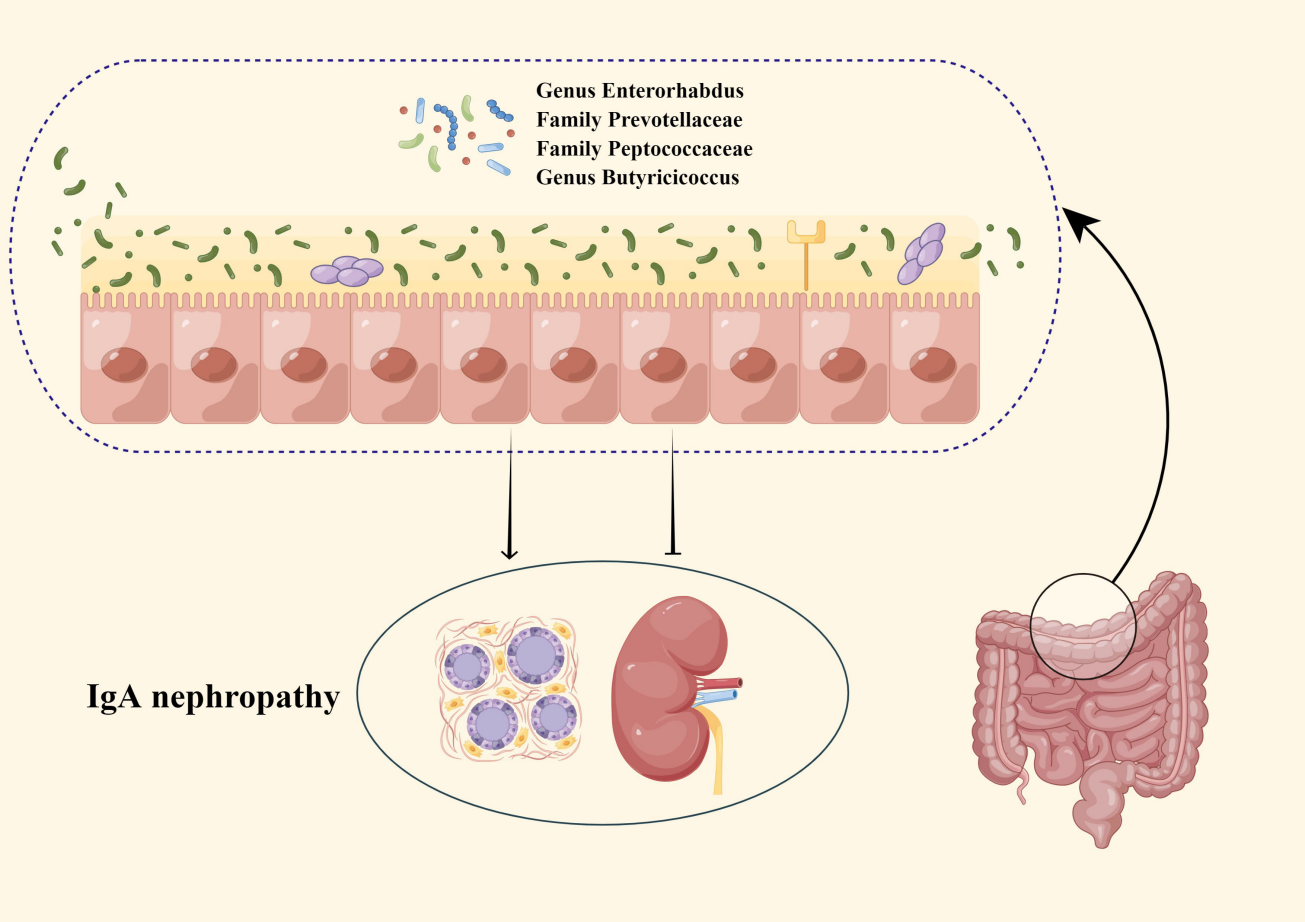
The present MR study reveals that GM may causally influences IgAN.

IgA nephropathy (IgAN), an immune complex-mediated glomerulonephritis (Wang et al., 2020), is the most common primary glomerular disease worldwide (Schena and Nistor, 2018). The nephropathy has a poor outcome and represents an important cause of end-stage renal disease (D'Amico, 2000). Recently, some researchers have pointed out that GM has correlations with the risk of IgAN (Coppo, 2015; Chemouny et al., 2019; Lauriero et al., 2021). Several studies (Ley et al., 2006; Camilleri et al., 2012; De Angelis et al., 2014; Coppo, 2015; Kåhrström et al., 2016; Chemouny et al., 2019; Lauriero et al., 2021) speculated that GM affects IgAN in three main ways: first, GM can enhance the intestinal mucosal barrier by promoting the attachment of intestinal epithelial cells and promoting the expression of mucin, in addition, GM can block the adverse effects of intestinal toxins and antigens on the kidney by promoting the secretion of antimicrobial peptide substances. Second, GM could regulate innate and adaptive immune responses by inducing intestinal epithelial cells to express Nucleotide binding oligodomain-like receptor 1 (NOD-1) and Toll-like receptors (TLRs) on the intestinal mucosal surface, which in turn promote the production and maturation of gut associated lymphocytes tissue (GALT). Third, when the intestinal mucosal barrier is damaged, uremic toxins produced by the GM translocate, which in turn increases the absorption of toxins into the blood, leading to the development of IgAN by both aggravating systemic inflammation and causing an increase in circulating Gd-IgA1. Additionally, there were studies showing (Bouskra et al., 2008) that the severity of IgAN can be improved by correcting the disturbed GM. The above information suggested that GM may be an essential factor affecting IgAN and a potential treatment for IgAN. In this study, we performed MR analysis to genetically validate the causal relationship between GM and IgAN as well as to identify new specific causal bacterial taxa that provide novel biological markers for predicting IgAN (**Figure 4**).

Our study revealed that Genus Enterorhabdus, Family Peptococcaceae, Family Prevotellaceae, and Genus Butyricicoccus reached nominal significance, and subsequent additional analyses indicated that the results for Genus Enterorhabdus and Family Butyricicoccus were significant. Furthermore, we validated the results that achieved nominal significance using the MR-BMA method and ranked the results according to MIP, which showed that Genus Enterorhabdus was the most critical factor influencing the incidence of IgAN.

In this study, Genus Enterorhabdus, Family Peptococcaceae, and Family Prevotellaceae were protective factors for IgAN. However, it has not been explored by traditional epidemiological studies on the relevance of Genus Enterorhabdus, Family Peptococcaceae, and Family Prevotellaceae to IgAN. We speculated that the protective mechanisms of these bacterial taxa against IgAN involve gene-microbiota interactions and butyrate.

Genetic human-leukocyte-antigen (HLA) variants are major determinants affecting the development of infectious and inflammatory diseases (He et al., 2020; Cao et al., 2022), and the 8.1 ancestral haplotype (AH8.1, one of the ancestral HLA haplotypes) is tightly associated with a variety of immune-mediated diseases (Thorsby and Lie, 2005). We speculated that HLA might influence the development of IgAN (Fellay et al., 2007). In a Genome-Wide Analysis (Candore et al., 2002), Feehally et al. indicated that the HLA region contains susceptibility alleles that are most likely to contribute to the development of IgAN, and the mechanism may be related to the abnormal immune response due to an imbalance in the antigen presentation process, and may also be related to the abnormal production of cytokines (IL-4, IL-10 and TGF-β1) (Fellay et al., 2007; Cao et al., 2008). Interestingly, previous studies (Feehally et al., 2010) have found that the pathogenicity of susceptibility genes is inconsistent in populations carrying susceptibility genes, and some studies have speculated that the pathogenicity of genes may be influenced by external factors, for example, the development of disease associated with AH8.1 is strongly related to alterations of the GM (Rausch et al., 2011; Yu et al., 2011), known as similar gene-microbiota interactions. Studies have indicated (Scher et al., 2013) that Genus Enterorhabdus, and Family Prevotellaceae are correlated with HLA, specifically that a higher abundance of Genus Enterorhabdus and Family Prevotellaceae repress transcription of 8.1 ancestral haplotype and in turn reduce the risk of IgAN (Kriegel et al., 2011).

In addition, some studies have pointed out the protective effect of Genus Enterorhabdus, Family Prevotellaceae, and Family Peptococcaceae on IgAN from other perspectives. Genus Enterorhabdus caecimuris can colonize and degrade mucin directly on the mucosal surface, thereby enhancing the intestinal mucosal barrier. Improved intestinal mucosal barrier function can more effectively prevent the infiltration of LPS into the circulatory system and activate TLR signaling, which inhibits the release of pro-inflammatory cytokines (Hov et al., 2015).

Butyrate also plays a vital role in the gut-kidney axis, as some studies (Horton et al., 2008; Ha et al., 2014) have shown that various bacterial species of Genus Enterorhabdus (E. caecimuris, E. mucosicola), as well as Family Prevotellaceae, are butyrate-producing bacteria. Butyrate affects the gut-kidney axis through several different mechanisms, such as its ability to replace damaged intestinal cells and to regulate the expression of tight junction proteins or mucus production to promote intestinal integrity (Stanford et al., 2020). Based on the above studies, a higher abundance of Genus Enterorhabdus, Family Prevotellaceae, and Family Peptococcaceae may reduce the risk of IgAN by producing more butyrate or directly enhancing the intestinal mucosal barrier. As a result, based on these mechanisms, modulating the abundance of Genus Enterorhabdus, Family Prevotellaceae, and Family Peptococcaceae using various modalities such as fecal transplantation therapy or the use of specific probiotics may be used as a potential therapy for IgAN.

Apart from that, our study also found that Genus Butyricicoccus was a risk factor for IgAN. There are no traditional epidemiological studies that have analyzed the correlation between Genus Butyricicoccus and IgAN. Nevertheless, one study (Mi et al., 2022) pointed out that Genus Butyricicoccus was significantly negatively correlated with inflammatory indicators, cystatin levels, and positively correlated with glomerular filtration rate. It is speculated that the reason is possibly the involvement of Genus Butyricicoccus in the synthesis of butyrate (Jiang et al., 2016; Morrison and Preston, 2016), which in turn affects renal function and the indicators related to renal function.

Besides the mechanisms of butyrate affecting IgAN mentioned previously, there are several pathways through which butyrate can produce cellular effects to influence the physiological functions of the kidney. (I) G protein-coupled receptor (GPCR) signaling pathway regulatory mechanism: studies (Gryp et al., 2021) have revealed that butyrate can activate Gpr41 (free fatty acid receptor 3, FFAR3) and Gpr43 (free fatty acid receptor 4, FFAR4), which are expressed in both the kidney and renal arteries, thereby affecting the metabolic and immune responses of the kidney (Pluznick et al., 2013; Han et al., 2022). In addition, butyrate inhibited the proliferation of glomerular thylakoid cells induced by several factors *via* GPCR and can also reverse the production of reactive oxygen species (ROS) and malondialdehyde (MDA) (Takeyasu et al., 1988). (II) Non-GPCR signaling pathway regulatory mechanism: butyrate can regulate the differentiation of T cells (e.g., Treg cells and Th17 cells) by regulating energy metabolism (Maslowski et al., 2009). Treg cells have the function of anti-inflammation and maintenance of autoimmune tolerance, which can inhibit the production of inflammatory factors and Gd-IgA1 and thus have a protective effect against the development of IgAN, while Th17 cells have the function of mediating inflammatory responses and autoimmune diseases. The dynamic balance between Treg cells and Th17 cells is closely related to the development of immune and inflammatory diseases. Specifically, butyrate increases the basal and maximal oxygen consumption rate (OCR) of naive CD4+ T cells (Kanda et al., 2021). It shifts their metabolic pattern toward oxidative phosphorylation, which in turn promotes the differentiation of Treg cells (Maslowski et al., 2009). Butyrate also decreased the expression of HIF-1α in naive CD4+ T cells, thereby inhibiting the differentiation of Th17 cells (Maslowski et al., 2009). Apart from the above influencing factors, butyrate can also modulate gene expression, regulate immune homeostasis and maintain Treg/Th17 homeostasis by inhibiting histone deacetylation (Sun et al., 2017; Huang et al., 2017). In particular, the promotive effect of butyrate on Treg cell proliferation is also associated with histone deacetylase (HDAC). Acetylation of histones activates gene transcription, whereas HDAC folds DNA around histones and inhibits gene transcription. Forkhead box P3 (FoxP3) is the signature transcription factor of Treg cells and plays an essential role in sustaining the differentiation and anti-inflammatory functions of Treg cells (Wen et al., 2021). As an HDAC inhibitor, butyrate induces histone h3 acetylation on the Foxp3 intron. It promotes Foxp3 expression in naive CD4+ T cells, facilitating the differentiation of naive T cells into Treg cells (Koh et al., 2016), which in turn inhibits the production of inflammatory factors and Gd-IgA1. All the above evidence suggests that Genus Butyricicoccus is a protective factor for IgAN, which is inconsistent with our findings. The reason might be due to the gene-gene interactions and gene-environment interactions.

Our study had the advantage that we presented the first large-scale comprehensive MR study to predict the causal relationship between GM and IgAN at the genetic level. In addition, our study was based on the latest large GWAS data and used a genetic prediction method to identify the causal relationship between specific bacterial taxa and IgAN. As well as applying the MR method, we also applied the MR-BMA method to rank the results of the MR method to identify the bacterial taxa which had the largest impact on IgAN. It is worth noting that our study has some limitations as well: (I) The data on GM was only classified above the genus level, and at a more specialized level (e.g., species or strain), we could not derive a causal relationship between them and IgAN. (II) As GWAS data contain European ancestry only, it was impossible to generalize the results of this study to other races. (III) Considering the biological plausibility and the multi-stage statistical process, the use of Bonferroni-Corrected test may produce false negative results. Therefore, we did not strictly follow the multi-corrected P value to screen the bacteria. (IV) The SNPs data contained in phylum, class, order, family, and genus may have heavy overlap, which may lead to the reproducibility of MR analysis results. (V) Subjective judgments of the authors may result in the creation of bias when using phenoscanner to remove confounding factors. Therefore, caution is still needed in the interpretation of the study results. (VI) Sample overlap may lead to inflated test results, but we expected the effect to be small since there was no known sample overlap. (VII) The results would have been more specific and accurate if GWAS had used more advanced shotgun metagenomic sequencing analyses. Further analyses based on larger studies with more advanced methods are therefore needed in the future to assess gut microbial features (e.g., species) and IgAN using the high-resolution data. (VIII) Abnormal values were observed in the leave-one-out analysis, nevertheless, no other statistical methods showed anomalies.

## 5 Discussion

In this study, a causal relationship between GM and IgAN was confirmed, which suggests that nephrologists should be cautious about the kidney function of patients with disordered intestinal flora when there is an increase or decrease in the abundance of specific bacteria. However, the inverse causality was not proven. Moreover, this study also derived specific bacterial taxa associated with IgAN, which may become novel biomarkers for IgAN in the future. Further studies on these bacterial taxa may help in the prevention and treatment of IgAN, which in turn provide theoretical support for the study of the gut-kidney axis.

## Data availability statement

The datasets presented in this study can be found in online repositories. The names of the repository/repositories and accession number(s) can be found in the article/**Supplementary Material**.

## Author contributions

Conceptualization: YZ and FR; methodology: FR and QJ; software: FR and QJ; formal analysis: FR and QJ; investigation: FR and YZ; resources: FR and QJ; data curation: FR, QJ, and XR; writing—original draft preparation: FR; writing—review and editing: FR; visualization: TL and QJ; supervision: TL and QJ; project administration: YZ, FR, and TL; funding acquisition: YZ. All authors contributed to the article and approved the submitted version.
